# Developing a procedure to extract chenodeoxycholic acid and synthesize ursodeoxycholic acid from pig by-products

**DOI:** 10.1016/j.heliyon.2023.e18313

**Published:** 2023-07-17

**Authors:** On You Kim, Seung Yun Lee, Da Young Lee, Sun Jin Hur

**Affiliations:** a Department of Animal Science and Technology, Chung-Ang University, 4726 Seodong-daero, Daeduk-myeon, Anseong-si, Gyeonggi-do 17546, Republic of Korea; b Division of Animal Science, Division of Applied Life Science (BK21 Four), Institute of Agriculture & Life Science, Gyeongsang National University, Jinju 52828, Republic of Korea

**Keywords:** Pig by-products, Chenodeoxycholic acid extraction, Ursodeoxycholic acid synthesis, Bile salt hydrolase enzymes

## Abstract

This study was conducted to develop simple methods for the extraction of chenodeoxycholic acid (CDCA) and synthesis of ursodeoxycholic acid (UDCA) from pig by-products. The enzymatic method, which uses bile salt hydrolase (BSH) enzymes to extract CDCA, was found to be more efficient than the chemical method. The chemical method, which uses pig by-products, resulted in UDCA amounts of 6.05 mg, 0.51 mg, 3.04 mg, and 1.26 mg in 100 g of the liver, stomach, small intestine, and large intestine, respectively. The amounts of UDCA synthesized/100 g through the chemical and enzymatic methods required to extract CDCA were 3.48 g and 2.22 g, respectively. The procedure developed in this study was simplified by three stages compared to the conventional chemical method of extracting CDCA. Moreover, this study provides a technique that improves the utilization of pig by-products.

## 1 Introduction

Recently, meat consumption has increased with rising economic income, thereby resulting in environmental pollution due to slaughter by-products. The slaughter by-products are better utilized better for cattle than pigs, which are disposed of because they require greater processing at higher costs than those associated with consumption [1]. Various studies have been conducted regarding the development of new materials from pig by-products to address this imbalance [2]. Bile acid, the main component of bile juice, plays an important physiological function in the liver and digestive tract. Various enzymes convert liver cholesterol to primary bile acid, which combines with amino acids, such as glycine and taurine, in the gallbladder, where it is stored as bile salt. Stored bile acid is secreted into the duodenum, where primary bile acid is converted into secondary bile acid through deconjugation by intestinal bacteria. Bile acids are mainly reabsorbed in the terminal ileum and then into the liver via enterohepatic circulation [3].

Ursodeoxycholic acid (UDCA) was first discovered in the bile acid of polar bears and first isolated from the bile of Chinese bears at the University of Oklahoma [4,5]. [6] identified its chemical formula. UDCA, which is more soluble than other bile acids, is useful because of its antagonism to hydrophobic bile acids. It is effective in treating and preventing liver dysfunction as well as digestive and skin diseases. Furthermore, it suppresses fat absorption and excretion through the biliary tract. However, an effective UDCA synthesis method has not been established because only small quantities have been extracted from bears; moreover, capturing them is not allowed because they are endangered animals [7]. Subsequently, various studies have been conducted to develop chemical or enzymatic methods using body micro-organisms to synthesize and extract UDCA. However, the effective extraction and synthesis of UDCA using pig by-products have not been extensively studied. Therefore, the purpose of this study was two-fold: 1) to develop a simpler and more stable method that can employ heat treatments at lower temperatures than the previous method; 2) to develop a method for extracting and synthesizing UDCA from pig by-products using the bile salt hydrolase (BSH) enzyme.

## 2 Materials and methods

### 2.1 Preparation of pig by-products

**Fig. 1:**
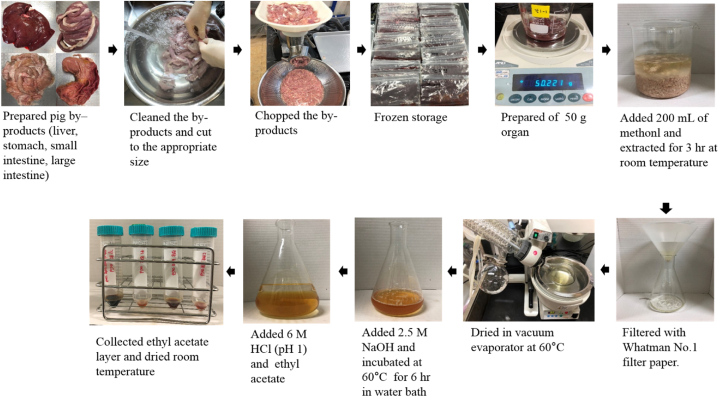
Preparation of by-products and extraction of bile acids.

Pig by-products, including the liver, stomach, small intestine, and large intestine, were separated at Dodram LPC (Anseong, Gyeonggi, Korea) and transported to the laboratory at ≤ 10 °C. After cleaning with running water (Fig. 1), the samples were ground using a grinder (Chopfer; MN-22S, Tech In Korea, Chungbuk, Korea) and stored at −30 °C until use. Pig gallbladders were obtained from the slaughtering house on the same day, frozen at −30 °C in Dodram LPC (Anseong, Gyeonggi, Korea), and transported to the laboratory. The BSH enzymes, used in this study to hydrolyze CDCA, were produced at Chung-Ang University using the *bsh* gene isolated from pig *Bifidobacterium thermophilum*. E*scherichia coli* DH5α was used as a host; pET22b(+)(Novagen, USA) was used as a cloning vector. Cloning into the NdeI-HindIII sites of pET22b resulted in the translational fusion of the bsh gene to the T7 promoter and *E. coli* ribosome binding site (RBS) of the plasmid. Plasmid pET22b was created in *E. coli* DH5α and transformed into *E. coli* BL21 to perform the overexpression studies. Luria–Bertani (LB) broth containing 50 μg/mL ampicillin and the modified fungus were cultured in a conical flask until the OD600 value was 0.5–1.0 at 37 °C. Subsequently, 100 mM isoprophylthiogalactopyranoside (IPTG) was added to induce BSH enzyme expression. The culture medium was centrifuged at 13 000 rpm for 10 min at 4 °C to recover the *Escherichia coli* DH5α. Afterward, the bacteria were dissolved in buffer solution (25 mM Tris-HCl (pH 8.3), 100 mM NaCl, 5% of glycerol) using a Sonicator (KFS-250, Korprotech, Korea) for 1 min, under an on/off (2/10) pulse with 58% amplitude, to obtain the supernatant, which was, then, centrifuged at 14 000 rpm for 20 min at 4 °C. Subsequently, the expression of BSH enzymes was confirmed using sodium dodecyl sulfate-polyacrylamide gel electrophoresis (SDS-PAGE). The BSH enzymes were isolated using a Ni + -NTA agarose column [[8], [9], [10]].

### 2.2 Extraction of bile acid

The process of bile acid extraction from pig by-products is shown in Fig. 1. Crude bile acid from pig by-products (liver, stomach, small intestine, and large intestine) was extracted using the methanol extraction method [10]. In the case of pig gallbladders, it was obtained directly and used. After adding 2500 mL of methanol (99.8%) to 500 g of pig by-products, the samples were homogenized at 10,000 rpm for 60 s using a homogenizer (SHG-15D, Scilab, Korea). The homogenized samples were refrigerated for 3 h and filtered using Whatman No. 1 filter paper to obtain crude bile acid from the minced pig by-products. A total of 50 g crude bile acid was dissolved in 500 mL of methanol (99.8%) and extracted using a water bath at 40 °C for 3 h.

The extracted samples were hydrolyzed for 6 h at 60 °C, after the addition of 50 mL of 2.5 M sodium hydroxide solution. Then, the 50 mL hydrolyzed samples were cooled to room temperature (25 °C), the pH was adjusted to 1 through the addition of dilute hydrochloric acid, and extraction was performed by adding 100 mL of ethyl acetate three times. After collecting the ethyl acetate layers, the bile acid was obtained using a rotary evaporator and frozen at −18 °C until use (Fig. 1).

### 2.3 Extraction of CDCA

CDCA was extracted from bile acid by two methods: chemical hydrolysis and biological hydrolysis.

#### 2.3.1 Chemical extraction of CDCA

**Fig. 2:**
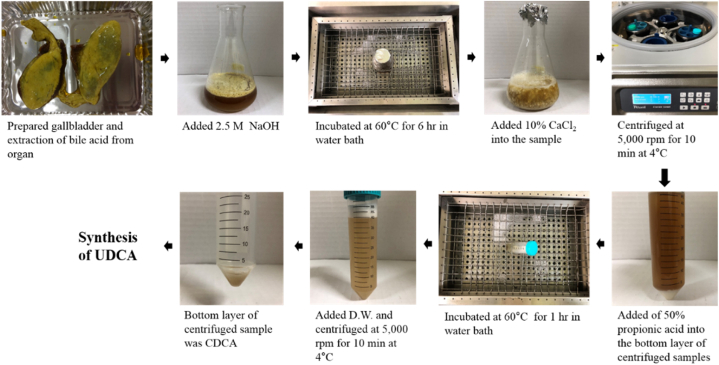
Chemical extraction of chenodeoxycholic acid (CDCA) and synthesis of ursodeoxycholic acid (UDCA).

CDCA was extracted by slightly modifying the methods of extraction from pig gallbladders and duck gallbladders [[11], [12], [13]]. A total of 10 mL sodium hydroxide (2.5 M) was added to the bile acid extracted from the pig by-products and the bile obtained from the pig gallbladders (samples: bile acid or bile; NaOH was 10:1, w/w). Next, these samples were hydrolyzed for 6 h at 60 °C in a water bath. Subsequently, 12 g of calcium chloride (Junsey, 99.9%) was added to the samples to obtain CDCA calcium salt sediments. Next, the CDCA calcium salt sediments were centrifuged at 5000 rpm for 10 min at 4 °C, and then, added to 10 mL of 50% propionic acid. The sediments were heated at 60 °C and dissolved, after which distilled water was added, and centrifugation performed at 5000 rpm for 10 min at 4 °C (Fig. 2).

#### 2.3.2 Enzymatic extraction of CDCA

Prior to extracting CDCA, standard substances, such as taurochenodeoxic acid (TCDCA) and glycochenodeoxic acid (GCDCA), were used to measure the separated BSH enzyme activity. Activation of the BSH enzyme decomposed GCDCA and TCDCA to UDCA, thereby reducing the GCDCA and TCDCA content. Using a slightly modified method from Ref. [14]; the experiment was conducted by dissolving 10 mM bile salt in 50 mM sodium phosphate buffer (pH 6.5) and diluting the BSH enzyme to 0.01% of the original solution. After 10 min in an incubator at 37 °C, 15% trichloroacetate was used to stop the reaction. BSH enzyme activity (9 units/mg), which one unit of BSH activity was defined as the amount of enzyme that liberated μmol of amino acids from the substrate per min, was conducted using high-performance liquid chromatography (HPLC); the conditions of which are shown in Table 1.

**Table 1:** High-performance liquid chromatography HPLC conditions for analyzing bile acids.

Parameter	Conditions
HPLC	Agilent 1100
Column	Fortis H_2_O C_18_ (150*4.6 mm id, 5 μm)
Injection volume (uL)	10
Mobile phase	70:30 (v/v) acetonitrile:phosphate acid buffer (pH 3)
Flow rate (min/mL)	1.0
UV detector (nm)	208
Column temperature (°C)	30
Run time (min)	10

**Fig. 3:**
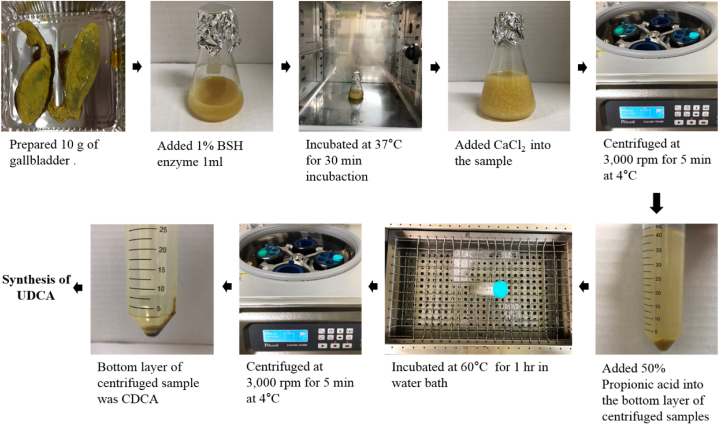
Extraction of CDCA from gallbladder and extraction of bile acid from organs.

To extract CDCA using biological hydrolysis enzymes, 1% BSH enzyme was added to bile acid extracted from pig by-products and bile obtained from pig gallbladders and incubated at 37 °C for 30 min. A total of 12 g of calcium chloride (Junsey, 99.9%) was added to form CDCA calcium salt sediments before centrifugation was performed at 3000 rpm for 5 min at 4 °C. Subsequently, the centrifuged samples were added to 10 mL of 50% propionic acid and incubated in a water bath at 60 °C for 1 h. Following, the samples were again centrifuged at 3000 rpm for 5 min at 4 °C before UDCA was synthesized (Fig. 3).

### 2.4 Synthesis of UDCA

**Fig. 4:**
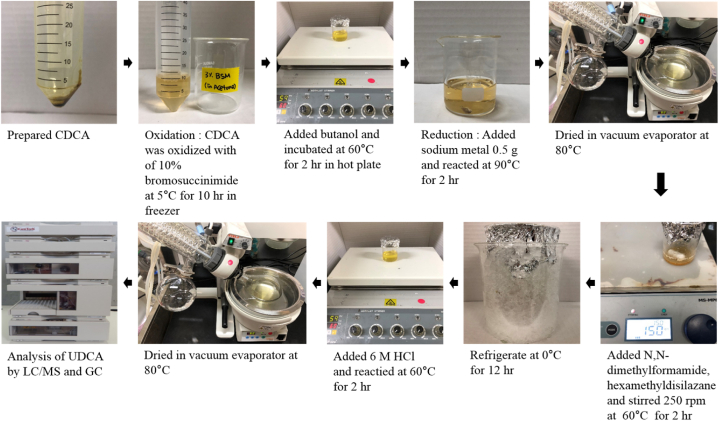
Synthesis of ursodeoxycholic acid (UDCA) from prepared chenodeoxycholic acid (CDCA).

UDCA was synthesized using a slightly modified oxidation and reduction method [15]. After acquiring the CDCA (precipitate) (Section 2.3.2), a total of 5 mL of 3% bromosuccinimide (Samchum, Korea, 99.9%), contained in acetone, was added to provide a 1:10, w/w ratio of CDCA precipitate:3% bromosuccinimide. This mixture was oxidized for 10 h at 5 °C to obtain an intermediate substance (7-ketolithocholic acid). A subsequent 2 h reaction was performed at 60 °C, after which 10 mL of tertiary butanol (Samchum, Korea, 99.5%) was added to the 7-ketolithocholic acid alongside 0.5 g metallic sodium, which is a catalyst that prevents a strongly exothermic reaction from occurring. It was handled in a dry area and added subdivide the metallic sodium to prevent a strong exothermic reaction. The mixture of CDCA and UDCA was synthesized by reduction. Next, 10 mL of dimethylformamide (DMF; Sigma, USA, 99.9%) and 1 mL of hexamethyldisilazane (HMDS; Sigma, USA, 99.9%) were added to the mixture and it was centrifuged (250 rpm) at 60 °C for 2 h (modified from Ref. [16]. Finally, the samples were stored at 0 °C for 12 h. The produced crystallized substances were dried at room temperature (25 °C). The samples were added to 1 mL of 6 M hydrochloric acid (Samchum, Korea, 36.45%) at 60 °C for 2 h, after which the reacted samples were dried in a vacuum evaporator and lyophilized to obtain the final UDCA crystals (Fig. 4).

### 2.5 Analysis of CDCA and UDCA by chromatography

HPLC (Agilent 1100) was employed to analyze the extracted bile acid, CDCA, and UDCA. Standard UDCA, TCDCA, GCDCA, cholic acid (CA), hyodeoxycholic acid (HDCA), and CDCA were purchased from Sigma-Aldrich (St. Louis, MO, USA). The extracted bile acid was dissolved in methanol and filtered using a 0.45 μm membrane filter and analyzed by HPLC; the conditions are shown in Table 1.

### 2.6 Liquid chromatography–mass spectrometry (LC/MS/MS)

The concentration analysis of CDCA and UDCA was conducted using LC/MS/MS (Thermo Scientific LTQ-Velos). The standard CDCA and UDCA used in the analysis were purchased from Sigma-Aldrich (St. Louis, MO, USA). The extracted bile acid was dissolved in methanol and filtered using a 0.22 μm membrane filter.

The LC/MS/MS analysis conditions are shown in Table 2. The MS-MS detector was measured at 3.5 kV in negative ionization mode at 250 °C using heated electrospray ionization (H-ESI II).

**Table 2:**
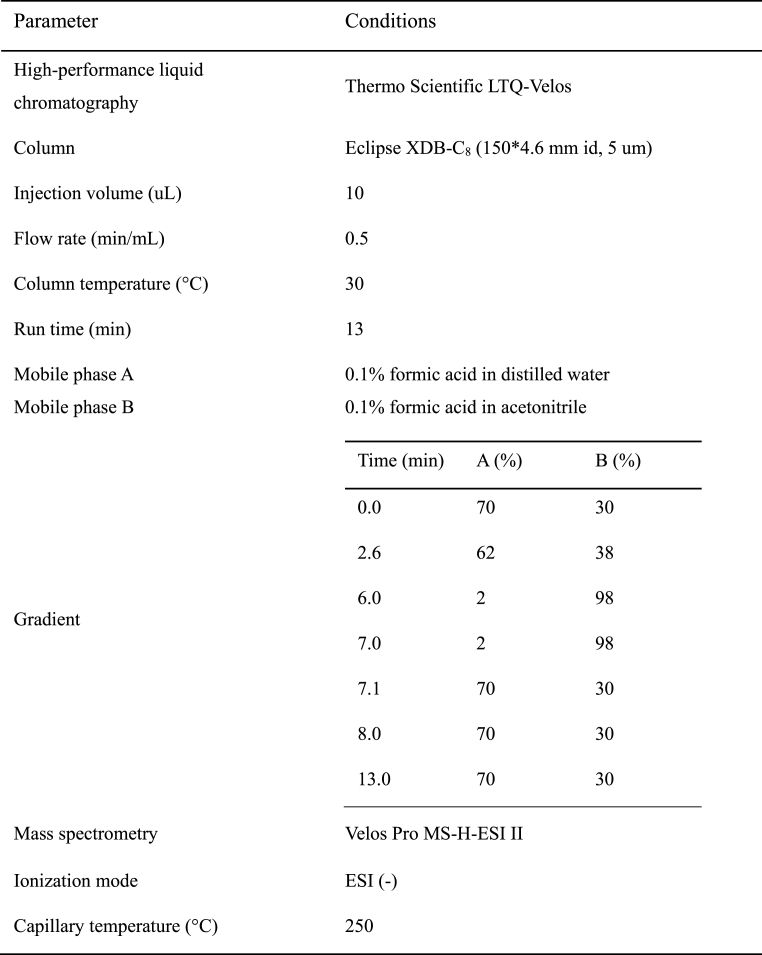
Liquid chromatography–mass spectrometry (LC/MS/MS) conditions for analyzing chenodeoxycholic acid (CDCA) and ursodeoxycholic acid (UDCA).

### 2.7 Statistical analysis

All the experiments were performed in triplicate. Sample data were analyzed for changes. The data that met assumptions of normality and homoscedasticity were analyzed using two-factor analysis of variance (ANOVA) using IBM SPSS Statistics for Windows, version 23.0 (IBM Corp., Armonk, NY, USA). The Student–Newman–Keuls multiple range test was used to determine significant differences (P < 0.05) between the mean sample concentrations values.

## 3 Results and discussion

### 3.1 By-product extracted bile acid components

**Fig. 5:**
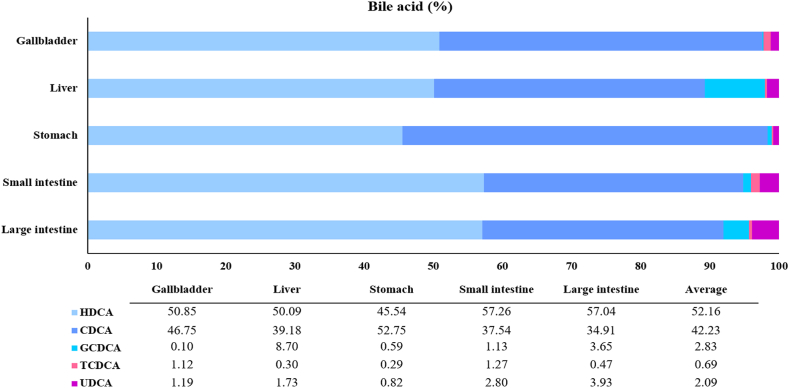
Quantities of bile acids in by-products. HDCA: hyodeoxycholic acid; CDCA: chenodeoxycholic acid; GCDCA: glycochenodeoxic acid; TCDCA: taurochenodeoxic acid; UDCA: ursodeoxycholic acid.

In previous studies, only bile acid composition was studied from pig gallbladders; however, in this study, the bile acid composition in various pig by-products (liver, stomach, small intestine, and large intestine) as well as in the gallbladder was measured [[17], [18], [19], [20]]. Bile acids produced in the liver are known as primary bile acids, while glycine and taurine are combined in the gallbladder to be concentrated and stored as bile salts (conjugated bile acids) [21]. Bile acids are bio-transformed to secondary bile acids in the large intestine by the gut microbiota [22]. Moreover, the formation of glycine-binding bile salts is 3-fold higher than taurine-binding bile salts. Indeed, the components of bile acid in pig by-products (liver, stomach, small intestine, large intestine, and gallbladder) were first measured and presented as relative content ratios for each organ (Fig. 5). HDCA comprised 50–57% of the components, while CDCA comprised 34–52%, and the UDCA content was highest in the large intestine. Additionally, the results were compared with subsequent experimental data. They were also used to compare the composite results of UDCA with those of a later CDCA extraction experiment. UDCA, which comprises the tertiary bile acids, is reabsorbed in the terminal ileum and colon before returning to the liver via the portal vein [22]. Fang et al. (2019) described the shift in serum, hepatic, and intestinal bile acid profiles in Large White pigs affected by heat stress, whereby chronic heat stress reduced the amount of taurine-binding bile acids in the serum and livers of these animals. Thus, heat stress can influence bile acid metabolism in pigs.

### 3.2 Extraction of CDCA

#### 3.2.1 Chemical extraction of CDCA from by-products

**Fig. 6:**
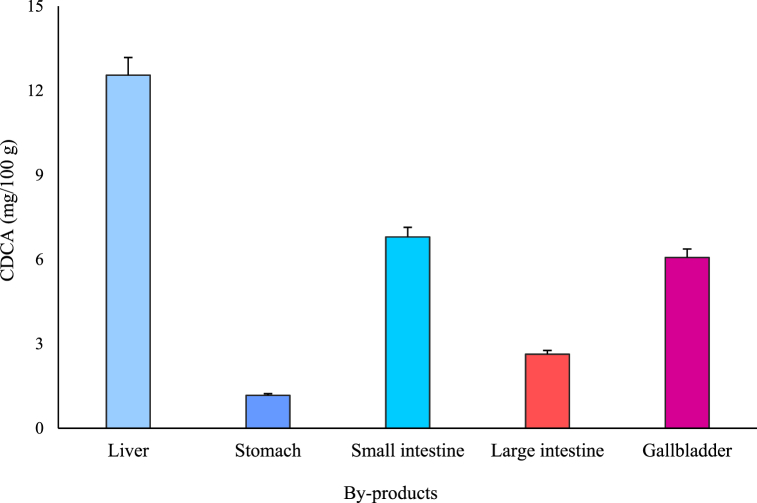
Quantities of chenodeoxycholic acid (CDCA) chemically extracted from by-products. Data are presented as mean ± SD (n = 3).

**Fig. 7:**
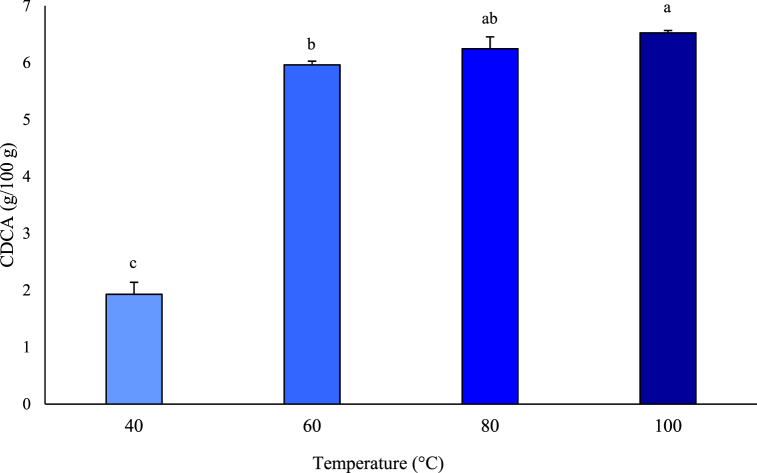
Changes in quantities of chenodeoxycholic acid (CDCA) chemically extracted from gallbladder, according to temperature. Data are presented as mean ± SD (n = 3). ^a, b, c^ Means within same parameter are significantly different (P < 0.05).

CDCA was extracted from by-products using a chemical extraction method at 100 °C to obtain the maximum yield. The amounts of CDCA extracted per 100 g from the liver, stomach, small intestine, and large intestine were 12.55 mg, 1.17 mg, 6.8 mg, and 2.63 mg, respectively. The CDCA content was highest in the liver since bile acid is synthesized there, while CDCA is secreted and absorbed in the small intestine. One study described how CDCA at relatively high concentrations stimulated SPX mRNA expression through FXR and RGR5 receptors in the mouse liver [23]. In contrast, 6.51 g of CDCA was extracted per 100 g of the gallbladder, where bile acid is stored (Fig. 6). Based on this result, the gallbladder was chosen for CDCA extraction and UDCA synthesis in the subsequent experiments since the overall CDCA concentration in the gallbladder (g/100 g organ) was high compared to other organs. Since the current research into how temperature affects CDCA extraction is limited, the CDCA extraction experiments were conducted using several heat treatment conditions, to first optimize a stable method. Therefore, the experiments were conducted at temperatures of 40–100 °C, in intervals of 20 °C. The lowest temperature of 40 °C was chosen owing to its closeness to the normal temperature for enzymatic extraction of 37 °C. Overall, the CDCA extraction quantities per 100 g were 1.93 g, 5.96 g, 6.25 g, and 6.51 g at 40 °C, 60 °C, 80 °C, and 100 °C, respectively (Fig. 7). There were no significant differences between the extraction quantities at 100 °C and 80 °C or those at 80 °C and 60 °C, while the CDCA content was significantly higher at 100 °C than that at 60 °C. However, considering commercial heat treatment stability, the difference in extraction amounts was not likely to be significant. Thus, 60 °C was determined as the most effective extraction temperature at which to perform CDCA extraction prior to UDCA synthesis.

#### 3.2.2 Enzymatic extraction of CDCA from by-products

**Fig. 8:**
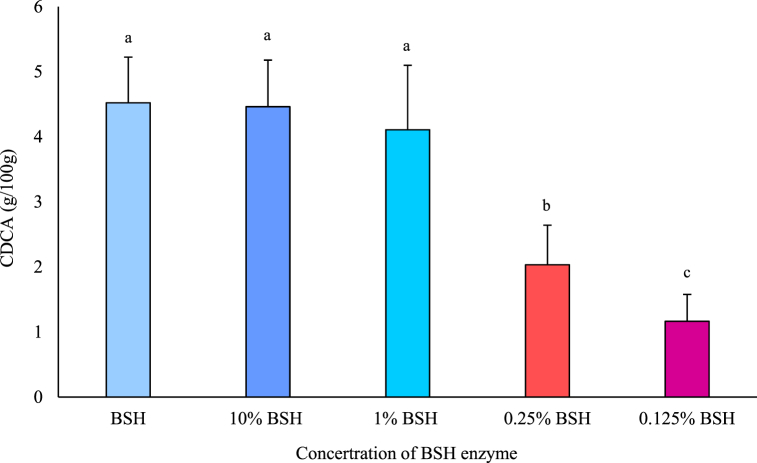
Changes in quantities of chenodeoxycholic acid (CDCA) enzymatically extracted from gallbladder according to bile salt hydrolase (BSH) enzyme concentration. Data are presented as mean ± SD (n = 3). ^a^, ^b^, ^c^ Means within same parameter are significantly different (P < 0.05).

Active testing and concentration setting experiments were conducted using GCDCA and TCDCA after the purification of BSH enzymes. Testing was conducted at a concentration of 10 mM, and the concentration of BSH enzymes in the original solution was determined until no activity remained. BSH enzymes showed significant activity differences depending on the refinement level (30–70%); the greatest activity was demonstrated at 0.125% concentration. Therefore, BSH enzyme concentrations ranging from the undiluted solution to 0.125% were set in the experiment. The results are shown in Fig. 8. The CDCA extract amounts per 100 g were 4.52 g, 4.46 g, 4.11 g, 2.03 g, and 1.16 g in the undiluted, 10%, 1%, 0.25%, and 0.125% BSH enzyme concentrations, respectively. CDCA extract amounts increased with rising concentrations of BSH enzymes, although there was no significant difference between BSH enzymes in the 1% and undiluted solution. Thus, CDCA extraction was the highest at a BSH enzyme concentration of 1%; thus, subsequent experiments were conducted using 1% BSH enzyme. BSH enzymes are choloylglycine hydrolases that are generated by gut microbiota and are responsible for catalyzing the hydrolysis of the amide-binding site of bile acid [24,25]. The BSH enzyme decomposes bile acids in the ileum and colon and has an affinity for taurine and glycine-conjugates. Notably, BSH enzymes have a higher activity towards hydrolyzing glycine-conjugated bile salts than taurine-conjugated bile acids [1]. Thus, BSH enzymes can be used to extract more CDCA in the presence of more glycine-conjugated bile salts than taurine-conjugated bile acids.

#### 3.2.3 Comparison of CDCA extraction methods

**Fig. 9:**
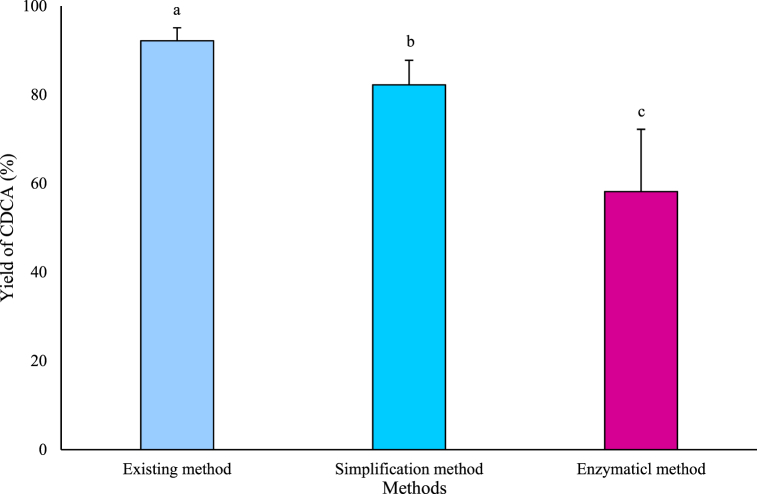
Extraction of chenodeoxycholic acid (CDCA) from gallbladder using different methods. Data are presented as mean ± SD (n = 3).^a^, ^b^, ^c^ Means within same parameter are significantly different (P < 0.05).

The CDCA yields were compared using three methods: i) the existing method with a hydrolysis temperature of 121 °C; ii) the simplification method with a hydrolysis temperature of 60 °C; iii) the enzymatic method using a BSH enzyme (Fig. 9). The CDCA yields from the existing and simplification methods were 92.09% and 82.24%, respectively; however, the enzymatic method produced a yield of 58.18%. Table 3 shows the previously used existing methods, which required 8 h of hydrolysis and 24 h of extraction as well as heat treatment at 121 °C. However, in this study, the heat treatment temperature and hydrolysis time was reduced to 60 °C and 6 h, respectively and the extraction stage was reduced by three steps. In addition, a lower temperature (37 °C) and reduced duration (2 h) were shown to produce CDCA with the enzymatic method.

**Table 3:** Extraction of chenodeoxycholic acid (CDCA) using different methods.

Factors	Existing method	Simplified method	Enzymatic method
Extraction time (h)	24	8	2
Hydrolysis reaction time (h)	8	6	1
Temperature (°C)	121	60	37
Reusability	X	X	Ｏ (Possibility)
Wastewater	Alkaline	Alkaline	X
Yield (%)	92.09 ± 3.13^a^	82.24 ± 5.91^b^	58.18 ± 14.03^c^

Data are presented as mean ± SD (n = 3).

^a, b, c^ Means within same parameter are significantly different (P < 0.05).

### 3.3 Synthesis of UDCA

**Fig. 10:**
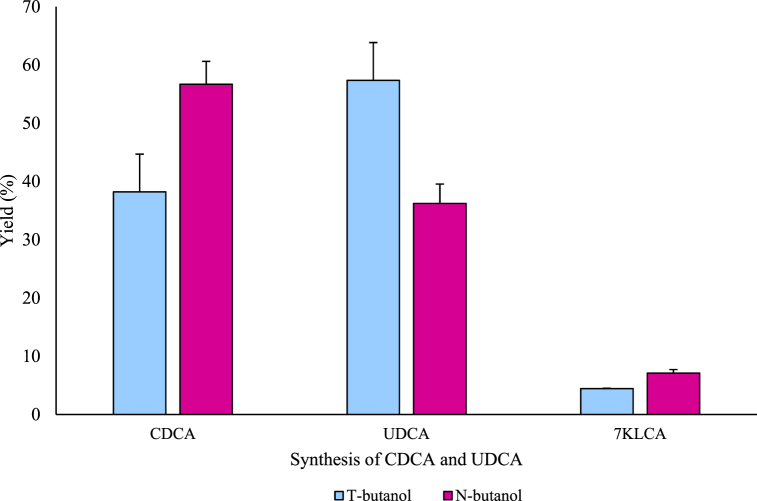
Yield of chenodeoxycholic acid (CDCA) and ursodeoxycholic acid (UDCA) mixture from extracted CDCA. Data are presented as mean ± SD (n = 3). KLCA: ketolithocholic acid.

**Fig. 11:**
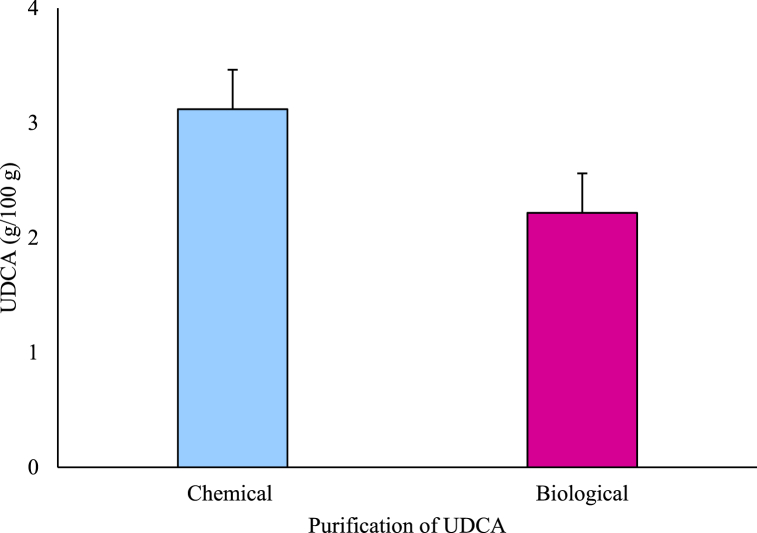
Purification of ursodeoxycholic acid (UDCA) in gallbladder. Data are presented as mean ± SD (n = 3).

UDCA was synthesized from CDCA extracted at 60 °C using organic solvents. In previous studies, several organic solvents were used for reduction during UDCA synthesis [[26], [27], [28]]. However, in this study, only two of the most effective organic solvents, namely, primary butanol and tertiary butanol were used for synthesis. The percentages of UDCA synthesized using primary and tertiary butanol were 36.23% and 57.34%, respectively (Fig. 10). This may be attributed to the higher electron affinity toward tertiary butanol than toward primary butanol. The tertiary group contains more alkyl groups compared to primary or secondary groups. Thus, the alkyl groups increase electron affinity, causing an increase in the donating electron and hydrogenation reaction by reduction [29]. Hence, tertiary butanol was selected as the reduction agent with which to synthesize UDCA. Performing hydrolysis using the chemical method produced a 6.07 g CDCA/100 g gallbladder extract, while the use of tertiary butanol obtained 3.48 g UDCA/100 g gallbladder extract. Comparatively, using the BSH enzyme in the biological method a 4.11 g CDCA/100 g gallbladder extract, while the use of tertiary butanol obtained 2.22 g UDCA/100 g gallbladder extract (Fig. 11). The refining rate was approximately 90%. Finally, hydrolysis at 60 °C and reduction using tertiary butanol were established as the optimal conditions for UDCA synthesis. CDCA extraction using BSH enzymes in the by-products did not yield UDCA because of their low activity and no CDCA extraction due to the use of organic solvents. From 100 g gallbladder tissue, 6.07 g CDCA was extracted (chemical extraction) by hydrolysis at 60 °C, and 3.48 g UDCA was synthesized using tertiary butanol. Using BSH enzymes, 4.11 g CDCA/100 g was extracted (hydrolytic extraction), and 2.22 g UDCA/100 g was synthesized. Although the UDCA yield (57.34%) was low, it could be increased by collecting the solution from the top during the refining process and proceeding with the oxidation–reduction process to again obtain 57% UDCA. Synthesizing using the enzymatic method presents low toxicity and no risk of explosion compared to the chemical methods since the method does not use metal ions; moreover, it can synthesize the desired bile acid. However, mass production using the enzymatic method remains difficult, with the limitation that the use of co-enzymes is required [30]. Therefore, by overcoming this obstacle, a simplified and effective enzymatic method can be used to increase the utilization of value-added materials for pig by-products.

## 4 Conclusions

In this study, the quantity and composition of bile acid in the liver, stomach, small intestine, and large intestine from pig by-products were determined. This study analyzed methods to extract CDCA from pig by-products and synthesize UDCA. Sources that can synthesize UDCA were found to contain bile acid including the gallbladder and other by-products, such as the liver, stomach, small intestine, and large intestine. The CDCA quantities were found to be highest in the order of gallbladder than in the liver, small intestine, large intestine, and stomach. The enzymatic method was developed and simplified to use the BSH enzyme to extract CDCA, which was a more efficient method compared to the previous chemical method. Moreover, using tertiary butanol during the reduction stage proved more efficient in synthesizing UDCA. The UDCA quantities from pig by-products were found to be highest in the liver and then descend in the order of small intestine, large intestine, and stomach. Furthermore, the UDCA amounts obtained were higher using the enzymatic method than from the chemical method. Thus, a method of synthesizing UDCA using a simplified hydrolysis procedure of CDCA in pig by-products was developed. Moreover, this study suggests that the enzymatic method, which uses the BSH enzyme, can be employed to obtain a more efficient extraction and synthesis of UDCA. Therefore, this study provides fundamental data for the future utilization of pig by-products.

## Ethics statement

The pig by-products were conducted according to the rules of the Institutional Animal Care and Use Committee of Chung-Ang University (IACUC approval number: A2022026).

## Author contribution statement

On You Kim: Conceived and designed the experiments; Performed the experiments; Analyzed and interpreted the data; Contributed reagents, materials, analysis tools or data; Wrote the paper.

Seung Yun Lee: Analyzed and interpreted the data; Contributed reagents, materials, analysis tools or data; Wrote the paper.

Da Young Lee: Analyzed and interpreted the data; Contributed reagents, materials, analysis tools or data.

Sun Jin Hur: Conceived and designed the experiments; Wrote the paper.

## Data availability statement

Data will be made available on request.

## Declaration of competing interest

The authors declare that they have no known competing financial interests or personal relationships that could have appeared to influence the work reported in this paper.
